# Comment on Lara Rzesnitzek (2013) “Early Psychosis” as a mirror of biologist controversies in post-war German, Anglo-Saxon, and Soviet Psychiatry

**DOI:** 10.3389/fpsyg.2013.00830

**Published:** 2013-11-08

**Authors:** Hanfried Helmchen

**Affiliations:** Klinik für Psychiatrie und Psychotherapie, Charité - Universitätsmedizin BerlinBerlin, Germany

**Keywords:** rzesnitzek, early psychosis, anglo-saxon, soviet, commentary

A mental illness with severe disturbances of subjective experiences and behavior with a progressive course, due to the onset of cognitive deterioration during the second and third decade of life, was described more than a century ago as dementia praecox (Kraepelin, 1896). In 1911 the diversity of existing marked symptoms led to the suggestion of a group of mental disorders, summarized as “Dementia praecox or group of schizophrenias” (Bleuler, 1911). Under the term schizophrenia various core symptoms of the diagnosis and/or of the disorder were defined, among others mainly: basic and secondary symptoms (Bleuler, 1911), first and second rank symptoms (Schneider, 1950), positive and negative symptoms (Andreasen, 1982). Sometimes the significance of the affective and intentional symptoms prevailed, at other times the cognitive disturbances were seen as the central phenomena, thus, e.g., in reframing schizophrenia as a “cognitive illness” (Kahn and Keefe, 2013).

For more than 100 years the enigma of schizophrenia has been under debate (Häfner, 2005). Particularly the nature of “basic” (Bleuler, 1911) or “negative” symptoms (Andreasen, 1982) is unclear: are these psychopathological phenomena—special cognitive disturbances, blunted affect sometimes difficult to differentiate from depressive disturbances (Häfner et al., 2013), and intentional disturbances—core symptoms of the disease or risk factors or consequences of the disease—or a mixture of all of them?

Lara Rzesnitzek (2013) in her informative and readable review recalls the early discussion on the nosological status of “early psychosis”: are its seemingly unspecific but in its entirety rather specific psychopathological phenomena before the manifestation of unequivocally psychotic symptoms dispositional and stable risk factors or initial symptoms of a gradually developing schizophrenia? Today's psychiatrists may wonder about the categorical black-or-white thinking of former conceptualists due to the currently dominating, more multiconditional concept, i.e., a specified bio-psycho-social model: developmental interactions between genetically conveyed sensitivity toward a distinct social context (and perhaps perinatal brain lesions as well) may form a disposition of vulnerability for critical life events, e.g., hormonal changes or social stress during adolescence (Zubin and Spring, 1977; Häfner, 2002; Haddad and Meyer-Lindenberg, 2012). “Early psychosis” today is subject to empirical long-term investigations on transition rates from bland symptoms to full blown psychoses, on contextual conditions and consequences, such as the Mannheim ABC-Study (Häfner et al., 2013), and on treatment.

In view of this I will comment on some ethical implications of “early psychosis,” irrespectively whether the symptoms indicate risk factors with predictive value or a beginning psychosis, because symptoms will be treated only if they intensify over time into functional or social handicaps. Due to the blandness of symptoms and their sluggish manifestation the diagnosis of “early schizophrenia” is difficult. This implies a particular *responsibility of the diagnostician* with regard to various aspects:

1. The diagnosis of the mental illness “psychosis” or even “schizophrenia” may stigmatize the concerned person, e.g., it may put a strain on the atmosphere in the family—and even may lead to a self-fulfilling prophecy in the sense of a disturbed mental development of the person involved.
  (In connection with the potential of psychiatric diagnoses to stigmatize their bearers it should be mentioned that in 2002 the Japanese Society of Psychiatry and Neurology substituted the term schizophrenia (“split-brain disorder”) with the neutral term “integration disorder,” in order to avoid a negative stigmatizing effect with the result of lowering the threshold for the contact of concerned persons with professionals (Sato, 2006). Together with this renaming Japanese psychiatrists also changed the etiological concept of schizophrenia from Kraepelin's biological disease concept to the vulnerability-stress model (Zubin and Spring, 1977) and thereby found it easier to explain the disorder to patients).
2. The uncertainty of diagnosis is open to other than medical influence, e.g., political influence, as was the case with dissidents in the former USSR who were silenced by a psychiatric diagnosis, particularly that of “sluggish schizophrenia,” in order to keep them away from the public in special psychiatric hospitals (Bloch and Reddawy, 1984; van Voren, 2010). However, not only misuse of psychiatric diagnoses has happened, but also their use in protecting patients, e.g., less stigmatizing terms for schizophrenia were used in the 1930s in National Socialistic Germany in order to protect patients from forced sterilization, which was demanded by law.

The diagnostic uncertainty of “early psychosis” and thereby its prognostic invalidity also calls for *the responsibility of the therapist*. A major discussion deals with the problem of preventive treatment (Klosterkotter et al., 2001). The chance of preventing a full blown psychosis must be contrasted with the risk of side effects of drug treatment in a person who never would have become psychotic without treatment, i.e., the risk of side effects of unnecessary treatment. However, the benefit-risk-estimation (Helmchen, in press) in such cases is difficult insofar as the psychiatrist:

1. must deal with a large degree of uncertainty of predictive criteria of “early psychosis,”
2. must consider the risk of stigmatization by a premature or unnecessary diagnosis for the concerned person, and
3. must explain understandably the probabilities of transition from “early psychosis” to full blown psychosis and of its prevention by treatment.

Corresponding to these demands the promotion of the concept of existential philosophy by anthropological psychiatrists was helpful, because it opened up an understanding of the subjective experiences of the (pre-)psychotic individual and fostered the recognition of the person and efforts to understand comprehensively the individual patient: the better the knowledge of a person in his/her contexts the better he/she can be informed appropriately.

However, Rzesnitzek's description exaggerates the role of the anthropological concept in West German psychiatry of the 1950s and 1960s, because it did not dominate the entire West German psychiatry but mainly the Frankfurt school of Jürg Zutt and Caspar Kulenkampff and, more or less, the southwest region of Germany. Furthermore, at the same time the very successful drug treatment of people with psychoses stimulated a new interest in neurochemistry, brain functions, and biological aspects of psychosis, and a network of young psychiatrists established long-term investigations on the course and treatment of schizophrenia. Thus, it was not a complete change from the biologically oriented nomothetic approach to a hermeneutic-idiographic concept, but rather the latter was an important addition to the former.

Two additional remarks may be helpful:

1. Today, terms such as the “schizophrenic person” or even “the schizophrenic” are no longer used, because they identify the mental illness schizophrenia with its bearer and thereby extend the negative stigma of the term schizophrenia to the concerned patient. Therefore, terms such as a “person with schizophrenia” comparable to “a person with a bone fracture” are preferred.
2. It might be misunderstood to translate the German term “Schub” as “phase” because the German term “Phase” was restricted to episodes of affective disorders. According to the dominating concept of the Kraepelinian dichotomy of “endogenous psychoses” at that time episodes of psychotic disorders were differentiated terminologically as “Schub” for schizophrenia and “Phase” for manic-depressive disorders. This terminology implied a course of affective disorders with completely remitting episodes, i.e., “Phasen,” but a progressive course of schizophrenia with a remaining residual on a lower level after an episode, i.e., “Schub” in the sense of taking a step downward. However, this terminology is no longer used, due to the fact that episodes of pure affective disorders may end with a remaining residual, and unequivocal episodes of schizophrenia may remit completely.
